# Diaqua­bis­(seleno­cyanato-κ*N*)bis­(pyrimidine-κ*N*)manganese(II)

**DOI:** 10.1107/S1600536810028941

**Published:** 2010-07-24

**Authors:** Mario Wriedt, Inke Jess, Christian Näther

**Affiliations:** a Institut für Anorganische Chemie, Christian-Albrechts-Universität Kiel, Max-Eyth-Strasse 2, 24098 Kiel, Germany

## Abstract

In the crystal structure of the title compound, [Mn(NCSe)_2_(C_4_H_4_N_2_)_2_(H_2_O)_2_], the manganese(II) cation is coordinated by two *N*-bonded pyrimidine ligands, two *N*-bonded seleno­cyanate anions and two *O*-bonded water mol­ecules in a distorted octa­hedral coordination mode. The asymmetric unit consists of one manganese(II) cation, located on a centre of inversion, as well as one seleno­cyanate anion, one water mol­ecule and one pyrimidine ligand in general positions. The crystal structure consists of discrete building blocks of composition [Mn(NCSe)_2_(pyrimidine)_2_(H_2_O)_2_], which are connected into layers parallel to (101) by strong water–pyrimidine O—H⋯N hydrogen bonds.

## Related literature

For a related pyrimidine structure, see: Lipkowski & Soldatov (1993 ▶). For general background to the use of thermal decomposition reactions for the discovery and preparation of new ligand-deficient coordination polymers with defined magnetic properties, see: Wriedt & Näther (2009*a* ▶,*b* ▶); Wriedt *et al.* (2009*a* ▶,*b* ▶).
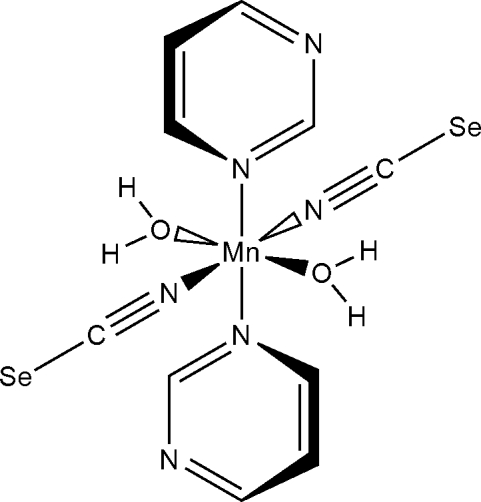


## Experimental

### 

#### Crystal data

[Mn(CNSe)_2_(C_4_H_4_N_2_)_2_(H_2_O)_2_]
*M* *_r_* = 461.12
Monoclinic, 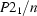

*a* = 9.2402 (7) Å
*b* = 9.6012 (6) Å
*c* = 10.2099 (8) Å
β = 111.505 (8)°
*V* = 842.74 (11) Å^3^
*Z* = 2
Mo *K*α radiation
μ = 5.11 mm^−1^
*T* = 170 K
0.10 × 0.07 × 0.04 mm

#### Data collection

Stoe IPDS-1 diffractometer
Absorption correction: numerical (*X-SHAPE* and *X-RED32*; Stoe & Cie, 2008 ▶) *T*_min_ = 0.653, *T*_max_ = 0.818
9472 measured reflections
2024 independent reflections
1795 reflections with *I* > 2σ(*I*)
*R*_int_ = 0.043

#### Refinement

*R*[*F* ^2^ > 2σ(*F* ^2^)] = 0.026
*wR*(*F* ^2^) = 0.064
*S* = 1.03
2024 reflections
98 parameters
H-atom parameters constrained
Δρ_max_ = 0.50 e Å^−3^
Δρ_min_ = −0.51 e Å^−3^

### 

Data collection: *X-AREA* (Stoe & Cie, 2008 ▶); cell refinement: *X-AREA*; data reduction: *X-AREA*; program(s) used to solve structure: *SHELXS97* (Sheldrick, 2008 ▶); program(s) used to refine structure: *SHELXL97* (Sheldrick, 2008 ▶); molecular graphics: *XP* in *SHELXTL* (Sheldrick, 2008 ▶); software used to prepare material for publication: *XCIF* in *SHELXTL* (Sheldrick, 2008 ▶).

## Supplementary Material

Crystal structure: contains datablocks I, global. DOI: 10.1107/S1600536810028941/bv2144sup1.cif

Structure factors: contains datablocks I. DOI: 10.1107/S1600536810028941/bv2144Isup2.hkl

Additional supplementary materials:  crystallographic information; 3D view; checkCIF report

## Figures and Tables

**Table:** 

Mn1—O1	2.1582 (14)
Mn1—N11	2.1840 (19)
Mn1—N1	2.3328 (18)

**Table:** 

O1—Mn1—O1^i^	180.0
O1—Mn1—N11	90.29 (7)
O1^i^—Mn1—N11	89.71 (7)
O1—Mn1—N1^i^	90.44 (6)
N11—Mn1—N1^i^	93.23 (7)
N11^i^—Mn1—N1^i^	86.77 (7)
O1—Mn1—N1	89.56 (6)

Symmetry code: (i) 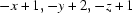.

**Table 2:** Hydrogen-bond geometry (Å, °)

*D*—H⋯*A*	*D*—H	H⋯*A*	*D*⋯*A*	*D*—H⋯*A*
O1—H2*O*1⋯N2^ii^	0.84	1.93	2.748 (2)	164

Symmetry code: (ii) 
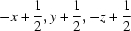
.

**Fig. 1.**
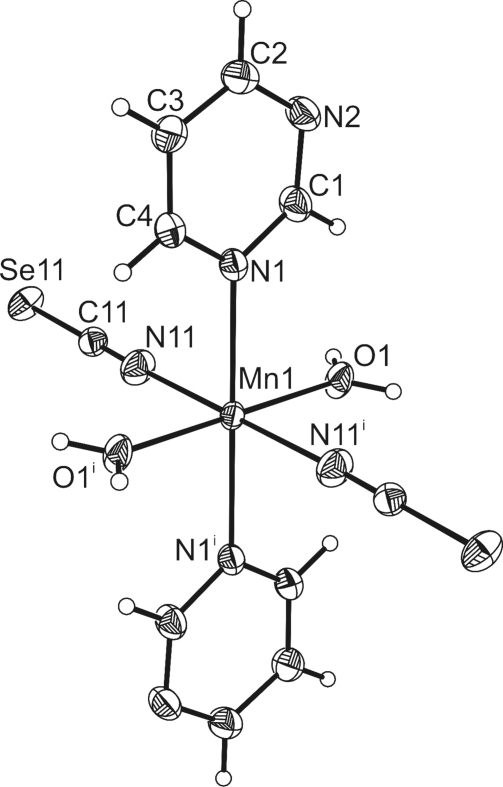
: Crystal structure of the discrete title compound with labelling and displacement ellipsoids drawn at the 50% probability level. [Symmetry codes: (i) -x+1, -y+2, -z+1.]

**Fig. 2.**
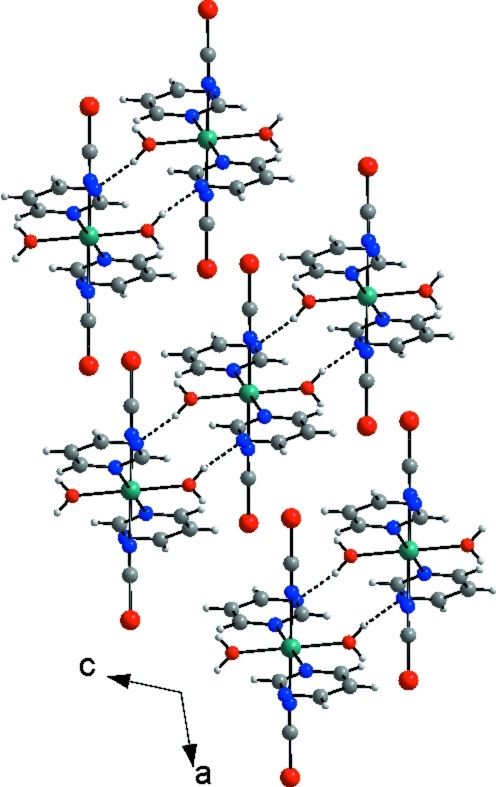
: Crystal structure of the title compound with view along the crystallographic b axis. The dashed lines indicate N···H—O hydrogen bonding.

**Fig. 3.**
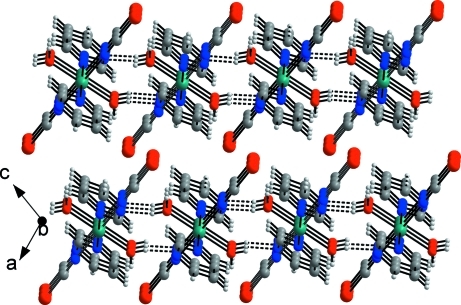
: Packing of two single layers with view approximately along the crystallographic b axis. The dashed lines indicate N···H—O hydrogen bonding.

## supplementary crystallographic information

### Comment

Recently, we have shown that thermal decomposition reactions are an elegante route for the discovering and preparation of new ligand-deficient coordination polymers with defined magnetic properties (Wriedt & Näther, 2009*a*, 2009*b*; Wriedt, Sellmer & Näther, 2009*a*, 2009*b*). In our ongoing investigation on the synthesis, structures and properties of such compounds based on paramagnetic transition metal pseudo-halides and N-donor ligands, we have reacted manganese(II) dichloride, potassium selenocyanate and pyrimidine in water. In this reaction single crystals were obtained, which were identified as the title compound by single-crystal X-ray diffraction.

The title compound of composition [Mn(NCSe)_2_(H_2_O)_2_(pyrimidine)_2_] (Fig. 1) represents a discrete coordination complex, in which the manganese(II) cation is coordinated by two selenocyanato anions, two water molecules and two pyrimidine ligands in an octahedral coordination mode. The MnN_4_O_2_ octahedron is slightly distorted with two long Mn–N_pyrimidine_ distances of 2.3328 (18) Å, two short Mn–NCSe distances of 2.1840 (9) Å and two short Mn—OH_2_ distances of 2.1582 (14) Å, while the angles around the metal center range between 86.77 (7)–93.23 (7) and 180° (Tab. 1). The coordination of the metal center is similar to that in a related structure (Lipkowski & Soldatov, 1993). In the crystal structure the single complexes are connected *via* strong N_pyrimidine_···H_water_ hydrogen bonds into layers (see Tab. 2), which are located in the crystallographic *a*/*c*-plane (Fig. 2 and 3). The shortest intra- and interlayer Mn···Mn distances amount to 7.2911 (5) and 9.3672 (5) Å, respectively.

### Experimental

MnCl_2_, KNCSe and pyrimidine were obtained from Alfa Aesar. 1 mmol (126 mg) MnCl_2_, 2 mmol (288 mg) KNCSe, 0.25 mmol (20 mg) pyrimidine and 3 ml water were reacted in a closed snap-vail without stirring. After the mixture was standing for several days at room temperature colorless block shaped single crystals of the title compound were obtained in a mixture with unknown phases.

### Refinement

All non-hydrogen atoms were refined anisotropic. The *O*—*H*-hydrogen atoms were located in difference map, where the bond lengths set to ideal values and were refined using a riding model. All other H atoms were located in difference map but were positioned with idealized geometry and were refined isotropic with *U*_eq_(H) = 1.2 *U*_eq_(C) of the parent atom using a riding model with C—H = 0.95 Å.

### Crystal data

**Table:**

[Mn(CNSe)_2_(C_4_H_4_N_2_)_2_(H_2_O)_2_]	*F*(000) = 446
*M**_r_* = 461.12	*D*_x_ = 1.817 Mg m^−^^3^
Monoclinic, *P*2_1_/*n*	Mo *K*α radiation, λ = 0.71073 Å
Hall symbol: -P 2yn	Cell parameters from 9472 reflections
*a* = 9.2402 (7) Å	θ = 2.6–28.0°
*b* = 9.6012 (6) Å	µ = 5.11 mm^−^^1^
*c* = 10.2099 (8) Å	*T* = 170 K
β = 111.505 (8)°	Block, colourless
*V* = 842.74 (11) Å^3^	0.10 × 0.07 × 0.04 mm
*Z* = 2	

### Data collection

**Table:**

Stoe IPDS-1 diffractometer	2024 independent reflections
Radiation source: fine-focus sealed tube	1795 reflections with *I* > 2σ(*I*)
graphite	*R*_int_ = 0.043
Phi scans	θ_max_ = 28.0°, θ_min_ = 2.6°
Absorption correction: numerical (*X-SHAPE* and *X-RED32*; Stoe & Cie, 2008)	*h* = −12→12
*T*_min_ = 0.653, *T*_max_ = 0.818	*k* = −12→12
9472 measured reflections	*l* = −13→13

### Refinement

**Table:**

Refinement on *F*^2^	Secondary atom site location: difference Fourier map
Least-squares matrix: full	Hydrogen site location: inferred from neighbouring sites
*R*[*F*^2^ > 2σ(*F*^2^)] = 0.026	H-atom parameters constrained
*wR*(*F*^2^) = 0.064	*w* = 1/[σ^2^(*F*_o_^2^) + (0.0376*P*)^2^ + 0.3681*P*] where *P* = (*F*_o_^2^ + 2*F*_c_^2^)/3
*S* = 1.03	(Δ/σ)_max_ = 0.002
2024 reflections	Δρ_max_ = 0.50 e Å^−^^3^
98 parameters	Δρ_min_ = −0.51 e Å^−^^3^
0 restraints	Extinction correction: *SHELXL97* (Sheldrick, 2008), Fc^*^=kFc[1+0.001xFc^2^λ^3^/sin(2θ)]^-1/4^
Primary atom site location: structure-invariant direct methods	Extinction coefficient: 0.0110 (14)

### Special details

**Table:**

Geometry. All e.s.d.'s (except the e.s.d. in the dihedral angle between two l.s. planes) are estimated using the full covariance matrix. The cell e.s.d.'s are taken into account individually in the estimation of e.s.d.'s in distances, angles and torsion angles; correlations between e.s.d.'s in cell parameters are only used when they are defined by crystal symmetry. An approximate (isotropic) treatment of cell e.s.d.'s is used for estimating e.s.d.'s involving l.s. planes.
Refinement. Refinement of *F*^2^ against ALL reflections. The weighted *R*-factor *wR* and goodness of fit *S* are based on *F*^2^, conventional *R*-factors *R* are based on *F*, with *F* set to zero for negative *F*^2^. The threshold expression of *F*^2^ > σ(*F*^2^) is used only for calculating *R*-factors(gt) *etc*. and is not relevant to the choice of reflections for refinement. *R*-factors based on *F*^2^ are statistically about twice as large as those based on *F*, and *R*- factors based on ALL data will be even larger.

### Fractional atomic coordinates and isotropic or equivalent isotropic displacement parameters (Å^2^)

**Table:**

	*x*	*y*	*z*	*U*_iso_*/*U*_eq_	
Mn1	0.5000	1.0000	0.5000	0.01759 (12)	
N1	0.4202 (2)	0.77991 (19)	0.54320 (18)	0.0208 (4)	
N2	0.3036 (2)	0.5623 (2)	0.45030 (19)	0.0289 (4)	
C1	0.3525 (3)	0.6901 (3)	0.4381 (2)	0.0265 (5)	
H1	0.3380	0.7208	0.3456	0.032*	
C2	0.3267 (3)	0.5183 (3)	0.5807 (2)	0.0298 (5)	
H2	0.2947	0.4271	0.5938	0.036*	
C3	0.3961 (3)	0.6023 (3)	0.6969 (2)	0.0299 (5)	
H3	0.4128	0.5705	0.7894	0.036*	
C4	0.4398 (3)	0.7336 (2)	0.6731 (2)	0.0261 (5)	
H4	0.4856	0.7940	0.7511	0.031*	
N11	0.7272 (2)	0.9064 (2)	0.5380 (2)	0.0300 (4)	
C11	0.8449 (2)	0.8544 (2)	0.5539 (2)	0.0214 (4)	
Se11	1.02886 (3)	0.77302 (3)	0.58075 (3)	0.02776 (10)	
O1	0.42680 (19)	0.94905 (18)	0.27927 (15)	0.0283 (3)	
H1O1	0.4782	0.9061	0.2392	0.042*	
H2O1	0.3448	0.9730	0.2134	0.042*	

### Atomic displacement parameters (Å^2^)

**Table:**

	*U*^11^	*U*^22^	*U*^33^	*U*^12^	*U*^13^	*U*^23^
Mn1	0.0178 (2)	0.0179 (2)	0.0143 (2)	0.00340 (15)	0.00258 (15)	−0.00073 (14)
N1	0.0200 (8)	0.0227 (9)	0.0175 (8)	−0.0008 (7)	0.0044 (7)	−0.0010 (6)
N2	0.0341 (10)	0.0270 (11)	0.0222 (9)	−0.0077 (8)	0.0061 (7)	−0.0039 (7)
C1	0.0300 (11)	0.0291 (12)	0.0185 (10)	−0.0042 (9)	0.0068 (8)	−0.0022 (8)
C2	0.0345 (12)	0.0246 (12)	0.0279 (11)	−0.0045 (9)	0.0086 (9)	0.0002 (9)
C3	0.0381 (12)	0.0285 (12)	0.0200 (10)	−0.0020 (10)	0.0070 (9)	0.0028 (9)
C4	0.0289 (11)	0.0253 (11)	0.0191 (9)	−0.0015 (9)	0.0030 (8)	−0.0021 (8)
N11	0.0230 (9)	0.0297 (11)	0.0367 (10)	0.0060 (8)	0.0101 (8)	0.0036 (8)
C11	0.0218 (9)	0.0218 (10)	0.0205 (9)	−0.0012 (8)	0.0078 (7)	0.0022 (7)
Se11	0.02287 (14)	0.02978 (16)	0.03597 (15)	0.00821 (9)	0.01709 (10)	0.00904 (9)
O1	0.0356 (8)	0.0300 (9)	0.0136 (6)	0.0116 (7)	0.0024 (6)	−0.0026 (6)

### Geometric parameters (Å, °)

**Table:**

Mn1—O1	2.1582 (14)	C1—H1	0.9500
Mn1—O1^i^	2.1582 (14)	C2—C3	1.383 (3)
Mn1—N11	2.1840 (19)	C2—H2	0.9500
Mn1—N11^i^	2.1840 (19)	C3—C4	1.372 (3)
Mn1—N1^i^	2.3328 (18)	C3—H3	0.9500
Mn1—N1	2.3328 (18)	C4—H4	0.9500
N1—C1	1.340 (3)	N11—C11	1.153 (3)
N1—C4	1.347 (3)	C11—Se11	1.798 (2)
N2—C1	1.329 (3)	O1—H1O1	0.8400
N2—C2	1.337 (3)	O1—H2O1	0.8400
			
O1—Mn1—O1^i^	180.0	C1—N2—C2	116.70 (19)
O1—Mn1—N11	90.29 (7)	N2—C1—N1	126.4 (2)
O1^i^—Mn1—N11	89.71 (7)	N2—C1—H1	116.8
O1—Mn1—N11^i^	89.71 (7)	N1—C1—H1	116.8
O1^i^—Mn1—N11^i^	90.29 (7)	N2—C2—C3	121.7 (2)
N11—Mn1—N11^i^	180.0	N2—C2—H2	119.2
O1—Mn1—N1^i^	90.44 (6)	C3—C2—H2	119.2
O1^i^—Mn1—N1^i^	89.56 (6)	C4—C3—C2	117.2 (2)
N11—Mn1—N1^i^	93.23 (7)	C4—C3—H3	121.4
N11^i^—Mn1—N1^i^	86.77 (7)	C2—C3—H3	121.4
O1—Mn1—N1	89.56 (6)	N1—C4—C3	122.4 (2)
O1^i^—Mn1—N1	90.44 (6)	N1—C4—H4	118.8
N11—Mn1—N1	86.77 (7)	C3—C4—H4	118.8
N11^i^—Mn1—N1	93.23 (7)	C11—N11—Mn1	177.7 (2)
N1^i^—Mn1—N1	180.00 (9)	N11—C11—Se11	179.4 (2)
C1—N1—C4	115.56 (19)	Mn1—O1—H1O1	127.1
C1—N1—Mn1	121.24 (15)	Mn1—O1—H2O1	128.3
C4—N1—Mn1	123.19 (14)	H1O1—O1—H2O1	104.5

Symmetry codes: (i) −*x*+1, −*y*+2, −*z*+1.

### Hydrogen-bond geometry (Å, °)

**Table:**

*D*—H···*A*	*D*—H	H···*A*	*D*···*A*	*D*—H···*A*
O1—H2O1···N2^ii^	0.84	1.93	2.748 (2)	164

Symmetry codes: (ii) −*x*+1/2, *y*+1/2, −*z*+1/2.
